# Quantifying Fluid and Function in Suboptimal Responders Switched From an Anti-vascular Endothelial Growth Factor (VEGF) to Faricimab

**DOI:** 10.7759/cureus.56652

**Published:** 2024-03-21

**Authors:** David Sutter, Abigail Anderson, Sheila Wheatley, Veeral Sheth

**Affiliations:** 1 Ophthalmology, Midwestern University Chicago College of Osteopathic Medicine, Downers Grove, USA; 2 Ophthalmology, University Retina and Macula Associates, Chicago, USA

**Keywords:** anti-vegf, vascular endothelial growth factor (vegf), matlab, neovascular age-related macular degeneration, ocular coherence tomography, faricimab, retina

## Abstract

Background

Anti-vascular endothelial growth factor (VEGF) injections have been successful in reducing vision loss from neovascular age-related macular degeneration, a leading cause of blindness. Due to the high treatment burden and suboptimal responses, switching to bi-specific faricimab treatment may lead to improved outcomes.

Methods

This retrospective chart review evaluated if suboptimal responders to anti-VEGF injections had better outcomes when switched to faricimab. Suboptimal responders were defined as patients with a history of >3 months of injections and the presence of fluid after ≥3 injections. The primary endpoints were best-corrected visual acuity, treatment interval, and fluid levels. Visual acuity measurements and optical coherence tomography were performed before each injection. The total fluid area (TFA) was measured using MATLAB 2023a (MathWorks, Natick, MA, USA).

Results

Nineteen eyes were included in the analysis. After three faricimab injections, average letters increased from 54.5 to 59.0 (SD: 15.3; p<0.05), and the injection interval was extended from 7.6 to 9.3 weeks (SD: 3.9; p<0.01) after four injections. Patients also experienced anatomical retinal changes, with a reduction in the TFA to 47.3% (p<0.005) after the second injection and a reduction in pigment epithelial detachment height to 82.3% (p<0.005) after one injection. The central subfield thickness was significantly reduced after the second injection (90.6% (SD: 17.6%)* *p<0.05).

Conclusion

Switching to faricimab after a suboptimal anti-VEGF response results in improvements in visual acuity, reduced treatment burden, and reduced fluid levels.

## Introduction

Age-related macular degeneration (AMD) is the leading cause of irreversible central vision loss in adults over 50 in the United States [1]. Neovascular AMD (nAMD) is an advanced form of macular degeneration characterized by dysregulated choriocapillary invasion through Bruch’s membrane into the outer retina. It is hypothesized that upregulated vascular endothelial growth factor (VEGF) and angiopoietin-2 (Ang-2) are instrumental in pathological angiogenesis and endothelial destabilization, respectively [2-4]. Corrupted vessel integrity enables lipid exudation, subretinal fluid (SRF) or intraretinal fluid (IRF) accumulation, hemorrhage, and eventually fibrosis, all of which may contribute to progressive vision loss.

Intraocular injection of anti-VEGF is the mainstay of nAMD treatment and has been successful in reducing vision loss by over 40% [5]. Despite its proven efficacy, anti-VEGF treatment carries a significant injection burden with current therapeutics, such as ranibizumab and aflibercept, requiring injections every four to eight weeks [6].

Faricimab, the most recent addition to the approved nAMD treatment arsenal, is a bi-specific monoclonal antibody with both anti-VEGF-A and anti-Ang-2 Fab regions that promotes vascular stability [7]. The phase III clinical trials TENYA and LUCERNE, which evaluated 6 mg of faricimab vs. aflibercept injected up to every 16 weeks in naïve nAMD patients, demonstrated non-inferiority with respect to letter recovery, as well as a reduction in central subfield thickness (CST), choroidal neovascular (CNV) size, and leakage. Notably, 80% of patients treated with faricimab maintained 16- or 12-week dosing intervals after one year, and after two years, 60% of patients treated with faricimab maintained a 16-week interval [8,9].

Additionally, the TRUCKEE study demonstrated the real-world efficacy and safety of faricimab across 14 sites in 39 treatment-naïve eyes and 337 previously treated eyes. The study found that patients who switched from a previous anti-VEGF injection had 25.3 µM (p<0.001) and 38.1 µM (p<0.001) reductions in CST after one and three injections, respectively. It also showed that fewer eyes had SRF, IRF, and pigment epithelial detachment (PED) after one injection of faricimab, and this trend was found to continue even after the third injection [10].

Despite meaningful fluid drying and letter recovery across these large cohorts, it is critical to analyze efficacy within vulnerable subpopulations. One of the most vulnerable is the suboptimal responder, characterized by an anti-VEGF injection history of >3 months and SRF or IRF occurring after three injections. Long-standing fluid accumulation can make it difficult to analyze or measure CST due to irreversible structural damage. This often correlates with a persistent, resistant deterioration in best-corrected visual acuity (BCVA). In this retrospective analysis, we sought to quantify BCVA, treatment injection intervals, and fluid drying in this unique group of suboptimal responders after switching from anti-VEGF therapy to faricimab injections.

## Materials and methods

This study was a retrospective chart analysis of patients with nAMD who were suboptimal responders to anti-VEGF treatment and were subsequently switched to faricimab treatment. A suboptimal responder was defined as a patient with an anti-VEGF injection history of >3 months and the presence of SRF or IRF after >3 anti-VEGF injections.

The study has been evaluated by the Sterling IRB institutional review board and deemed not to require ethics approval. Patients were not involved in the design and conduct of this research.

Patients included in this study were treated at the University Retina, Chicago, IL, between February 2022 and May 2023 and satisfied the following inclusion criteria: (1) diagnosed with nAMD; (2) switched from a previous anti-VEGF to faricimab; and (3) judged to be suboptimal responders.

The primary objective of the study was to provide a quantitative measurement of the effect of faricimab on SRF and IRF in patients with nAMD who had a suboptimal response to anti-VEGF treatment. Endpoints included BCVA, injection interval, and anatomical changes in the fluid. Fluid levels in the eyes were further subcategorized into SRF or IRF.

Demographic characteristics and treatment history (number of previous injections, medication, and injection frequency) were collected. Visual acuity measurements and spectral domain optical coherence tomography (OCT) images were completed before each faricimab injection and analyzed using MATLAB 2023a (MathWorks, Natick, MA, USA) for the fluid area at each level of the OCT fast scan. The baseline was defined as the measurements collected on the day of the first faricimab injection before the injection was done.

With custom MATLAB software, the perimeter of each fluid pocket at each level of the OCT was identified, and the pixels within this area were summed. Total fluid area (TFA) was defined as the fluid area in pixels in the OCT taken on the day of injection. TFA represents total retinal fluid accumulation. The presence of retinal fluid (SRF or IRF), CST, and PED height (measured via OCT) were determined by each investigator. PED height was measured via HRA/Spectralis Viewing Module version 6.16.11.0 (Heidelberg Engineering, Franklin, MA, USA).

Baseline characteristics were tabulated as means and percentages and reported with standard deviations (SD). BCVA was converted to Snellen logMAR estimation. Changes in fluid were percent reductions from 100% (baseline). Significance values were determined using a two-tailed t-test and a two-way ANOVA, with a significance value of p<0.05 for all analyses. Snellen visual acuity was converted to the Early Treatment Diabetic Retinopathy Study (ETDRS) BCVA scoring using the formula ETDRS = 85 + 50 × log10 (Snellen fraction).

## Results

In total, 19 eyes from 17 patients were identified for inclusion: seven with SRF, nine with IRF, and three with SRF + IRF, of which 16 eyes were switched from aflibercept, one from ranibizumab, one from brolucizumab, and one from bevacizumab (Table 1).

**Table 1:** Baseline demographics IRF: intraretinal fluid, SD: standard deviation, SRF: subretinal fluid, VEGF: vascular endothelial growth factor The data has been represented as number (n), percent (%), and mean +/- SD

	Anti-VEGF to faricimab n=19 eyes
Age (years), mean (SD)	78 (10)
Sex, n (%)	
Male	11 (58.0%)
Female	8 (42.1%)
Patients with fluid, n (%)	19 (100%)
SRF	7 (37.8%)
IRF	9 (47.4%)
SRF + IRF	3 (15.8%)
Duration of anti-VEGF treatment (days), mean (SD)	1418 (1006.1)
Most recent treatment, n (%)	
Aflibercept	16 (84.2%)
Ranibizumab	1 (5.3%)
Brolucizumab	1 (5.3%)
Bevacizumab	1 (5.3%)
Baseline interval, weeks (SD)	7.6 (2.8)
Interval between 3^rd^ and 4^th^ faricimab injection, weeks (SD)	9.3 (3.9)
Fluid drying at 3^rd^ injection, n (%)	
≥99%	7 (36.8%)
≥50% to 98%	7 (36.8%)
<50%	5 (26.3%)
Letter increase at 3^rd^ injection	
≥5	7 (36.8%)
1-5	4 (21.0%)
No improvement	8 (42.1%)

**Figure 1:**
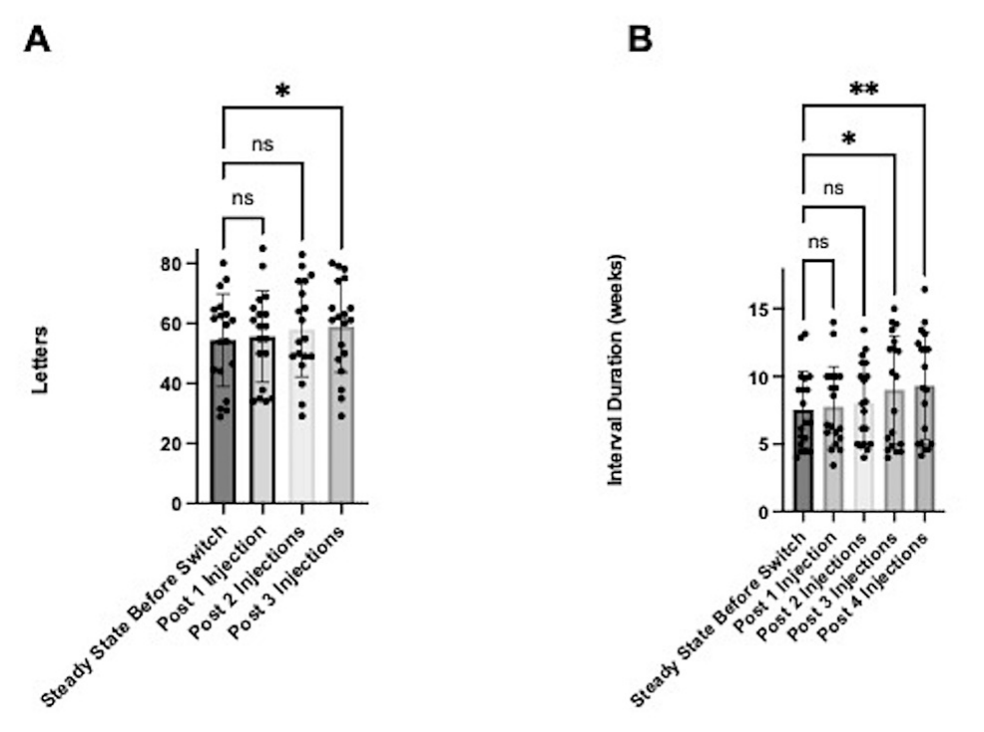
Functional changes in eyes with all fluid types (n=19) at one, two, and three injections relative to steady state. The data has been represented in mean +/- SD (A), letters (B), and interval duration (weeks). *p<0.05, **p<0.01 ns: not significant

Suboptimal responders who switched to faricimab incurred functional changes in their vision and injection interval extension (Figure 1). After receiving three faricimab injections, average letters increased from 54.5 (SD 15.5) to 59.0 (SD 15.3; p<0.05) (Figure 1a). After three faricimab injections, the injection interval was extended from 7.6 weeks (SD 2.8) to 9.0 weeks (SD 3.92; p<0.05). After four faricimab injections, the injection interval was extended to 9.3 weeks (SD 3.9; p<0.01; Figure 1B).

**Figure 2:**
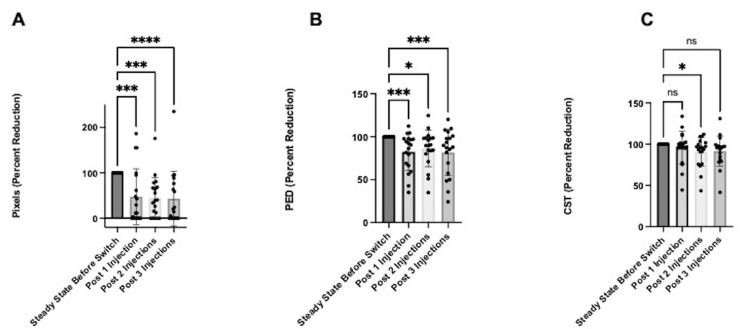
Anatomic changes in eyes with all fluid types (n=19) at one, two, and three injections relative to steady state. The data has been represented in mean percent reduction +/- SD (A) TFA in pixels, (B) PED, and (C) CST *p<0.05, **p<0.01, ***p<0.005, ****p<0.001 CST: central subfield thickness, PED: pigment epithelial detachment, TFA: total fluid area reduction

Additionally, suboptimal responders who switched to faricimab incurred anatomic retinal changes as early as eight weeks after the first injection (Figure 2). After the first faricimab injection, TFA was reduced to 47.3% (SD 61.6%; p<0.005), 44.8% (SD 44.7%; p<0.005) after two injections, and 43.1% (SD 60.4%; p<0.001; Figure 2A) after the third injection post-switch. On average, PED was reduced to 82.3% (SD 21.7%; p<0.005) after one faricimab injection, 86.3% (SD 21.2%; p<0.05) after two injections, and 81.6% (SD 26.8%; p<0.005) after three injections post-switch (Figure 2B). However, the change in mean CST was only significant after the second injection (90.6% (SD 17.6%), p<0.05; Figure 2C).

**Figure 3:**
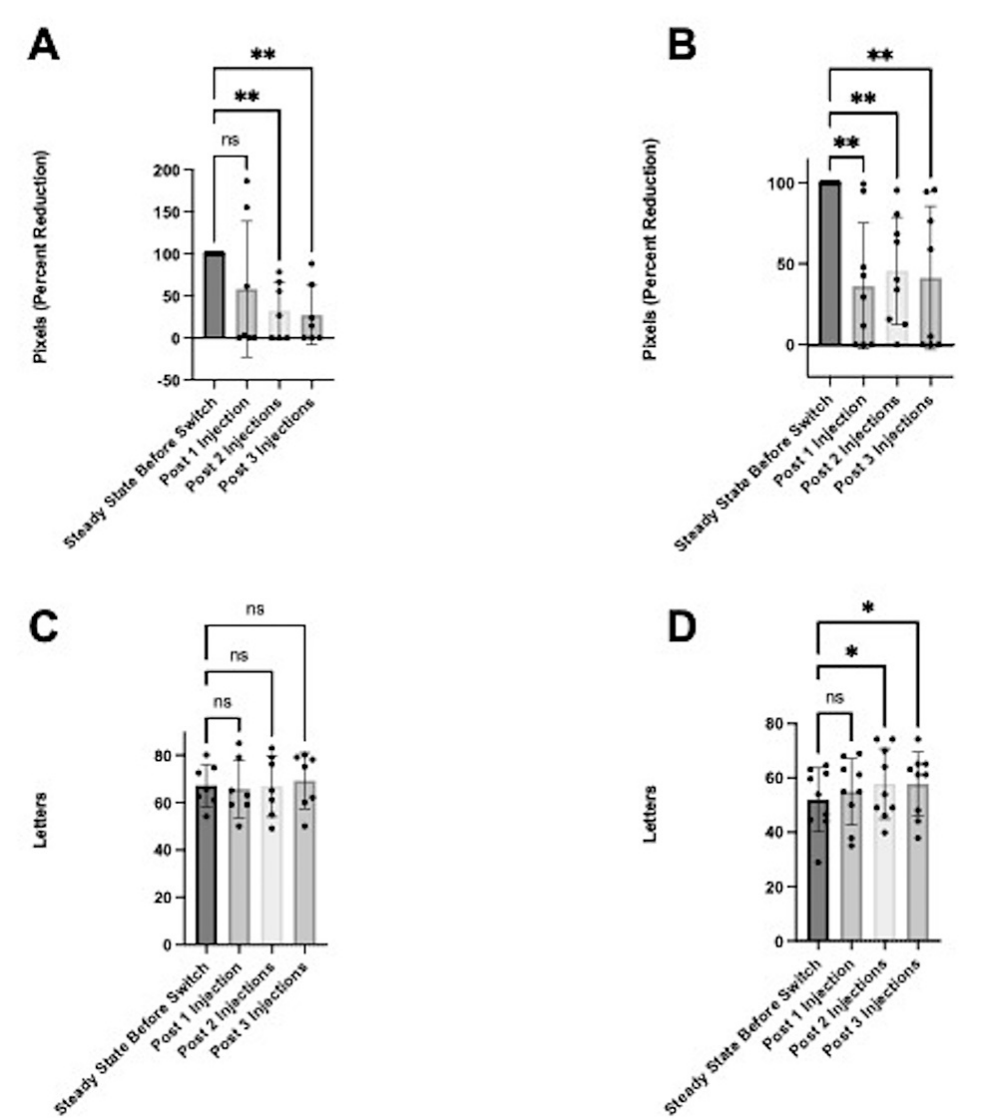
Comparing anatomy and function between fluid subtypes. The data has been represented in mean percent reduction +/- SD in TFA at one, two, and three injections relative to steady state in (A) eyes with SRF (n=7) and (B) eyes with IRF (n=9). The data has been represented in mean change in letters +/- SD at one, two, and three injections relative to baseline in (C) eyes with SRF (n=7) and (D) eyes with IRF (n=9) *p<0.05, **p<0.01 IRF: intraretinal fluid, PED: pigment epithelial detachment, SRF: subretinal fluid, TFA: total fluid area reduction

Suboptimal responders were further categorized into those with SRF or IRF, and the impact of faricimab on anatomy and function was examined. Due to the physiologic difference in fluid type between SRF and IRF, the impact of switching to faricimab was examined on these subpopulations’ anatomy and function (Figure 3). There was a significant reduction in TFA after the second and third faricimab injections in eyes with SRF (second injection: 32.4% (SD 34.1%), p<0.01; third injection: 27.2% (SD 35.2%), p<0.01; Figure 3A). Additionally, there was a significant reduction in TFA by the first, second, and third faricimab injections in eyes with IRF (first injection: 36.2% (SD 39.1%), p<0.01; second injection: 45.59% (SD 33.12%), p<0.01; third injection: 41.3% (SD 44.3%), p<0.01; Figure 3B).

In eyes with SRF, there was an increase in letters after each faricimab injection; however, none reached statistical significance (Figure 3C). In eyes with IRF, there was a significant increase in letters from baseline (51.6 (SD 12.6)) after the second (58.3 (SD 13.8); p<0.05) and third faricimab injections (57.3 (SD 12.5); p<0.05; Figure 3D).

## Discussion

This retrospective analysis of patients with nAMD with a suboptimal response to anti-VEGF treatment was conducted to see if patients who were switched to faricimab had improvements in BCVA, injection intervals, and fluid drying. The results support that switching to faricimab encourages the gain of letters, the ability to treat and extend, and fluid drying.

Anatomy drives clinical judgment when treating patients with nAMD. While CST has been shown to be a poor surrogate for visual acuity in nAMD, it is a clinically useful metric in assessing fluid drying and is relied upon heavily, especially in treat-and-extend protocols [11]. While real-world studies have used CST to show that faricimab reduces the number of patients with persistent fluid, this study quantifies the drying ability of faricimab in pixels in individual patients with either SRF or IRF.

In the cohort of suboptimal responders examined here, we observed a profound reduction in fluid pockets with faricimab, despite CST reductions only being significant after the second injection. In this subpopulation of nAMD patients with long-standing fluid, CST may not reflect clinically relevant fluid drying; TFA may serve as a more anatomically sensitive measurement in directing treatment. Using custom MATLAB software, we calculated the number of pixels in these fluid pockets, labeled TFA. By quantifying the fluid area in each slice of the OCT, we produced an endpoint that was more sensitive to clinically appreciable fluid drying. Following the switch to faricimab, 14 out of 19 eyes (74.0%) had a ≥50% reduction in TFA when compared to baseline. The clinical accuracy of TFA reduction is supported here by the concomitant PED reductions, appreciated as early as the first injection after the switch and maintained beyond the third injection post-switch.

In this study, we noted amplified TFA changes compared to CST depending on how the patient tolerated a treat-and-extend protocol. One patient, following a fluid reduction of 70% after their first injection of faricimab, missed the next appointment, leading to a 15-week interval between the first two injections. While their CST increased by 10%, TFA increased by 50%. After getting back on schedule, the patient’s fluid was reduced by the fourth injection. In another case, a patient saw near-complete fluid drying or 100% TFA reduction by the third injection, which was mirrored by only a 6% reduction in CST. This successful fluid drying warranted a three-week interval extension and showed that monitoring TFA with CST can support extending the injection interval versus just using CST alone.

Additionally, utilizing TFA as an endpoint reflects anatomical changes not appreciated by CST alone, especially in unconventional groups like suboptimal responders. Using this endpoint when comparing the efficacy of faricimab to other treatment paradigms may reveal insights that have not been previously considered.

Among previously treated eyes with either SRF or IRF, switching to faricimab encouraged significant letter recovery. Thirteen out of 19 eyes (68.4%) in this patient cohort had the ability to see more letters after the third injection, and seven out of 19 eyes (36.8%) improved by one full line. We also show that by the fourth injection after the switch, the injection interval as a group is significantly extended by two weeks.

Patients with either SRF or IRF underwent a significant reduction in TFA after the second and third injections. Interestingly, the IRF but not the SRF patient group showed a statistically significant increase in calculated letters by the second injection. Several studies that have probed the influence of SRF vs. IRF on visual outcomes in nAMD suggest that IRF is linked to lower visual acuity at baseline, correlating IRF to greater retinal toxicity [12-15]. Our results are in line with these findings, with IRF having baseline calculated letters of 51.6 (SD 12.6), while patients with SRF could see 67.3 (SD 9.0) letters at baseline. Following IRF drying, these eyes gained more than one full line of letters by the second and third injections on average after the switch. The consensus literature also supports that visual acuity is more stable despite SRF drying [13]. Our results show that despite a mean SRF reduction to 32.4% (SD 34.1%) by the second injection post-switch, there was a mean of 0 letters gained. Additionally, despite the SRF reduction to 27.2% (SD 35.2%) by the third injection post-switch, on average, patients gained 0 letters. While this mechanism is not precisely clear, it may likely be due to epithelial versus interstitial edematous damage.

Our analysis has several limitations, including that it is a retrospective analysis, and as such, the data were not collected with our specific research questions in mind, and there is a potential for missing or incomplete data. It is possible that important clinical variables may not have been consistently documented across all patient records. This inconsistency can introduce biases and make it challenging to draw definitive conclusions. Additionally, the absence of a control group limits our ability to establish causality between observed outcomes and specific treatments or disease characteristics. Furthermore, our findings are limited by the fact that they are drawn from a single clinical setting, potentially affecting the generalizability of the results to broader populations. Moreover, our small sample size may limit the representation of the broader population from which it was drawn, and individual variations may have had an amplified impact on the group. Despite these limitations, the insights gained from this study provide valuable observational data about switching suboptimal responders to faricimab treatment and contribute to a deeper understanding of nAMD management.

## Conclusions

This retrospective chart analysis shows that patients who are suboptimal responders to anti-VEGF injections may have an improved response if switched to faricimab. The patients analyzed here showed improvements in visual acuity, a reduction in treatment burden with the ability to treat and extend, and improvements in fluid drying.
